# Surgical treatment results and pathological features in pediatric occult tight filum syndrome

**DOI:** 10.1186/2045-8118-12-S1-P21

**Published:** 2015-09-18

**Authors:** Jeff Julian, Michael Punsoni, John Donahue, Ed Stopa, Petra M Klinge

**Affiliations:** 1 Neurosurgical Department, Rhode Island Hospital, USA; 2 Neuropathology, Rhode Island Hospital, USA; 3 Neuropathology, Rhode Island Hospital, USA; 4 Neuropatholgy, Rhode Island Hospital, USA; 5 Neurosurgical Department, Rhode Island Hospital, USA

## Introduction

Occult tight filum terminale syndrome (OTCS) is defined as a clinical syndrome of tethered cord and without “classic” radiographic evidence of low lying conus and/or fatty filum.

## Methods

A consecutive series of 11 children (2-17 years) diagnosed with tethered cord syndrome (Triad of neurological, urological and orthopedic findings) since 2010, a non-diagnostic MRI, underwent microsurgical resection of the filum. Presenting symptoms and symptoms most responsive to surgery, imaging and pathology of the filum were analyzed.

## Results

OTSC show the overall improvement in all dimensions of the clinical syndrome, e.g. scoliosis, walking and falling spells, incontinence and overall activity level due to improved pain. Increased tone in the lower extremities and foot deformities appeared as a negative predictor of improvement. Associated syringohydromyelia did not show any change in the 1 year follow-up MRI despite marked clinical improvement. Pathology shows a variety of features including “nerve twigs”.

## Conclusions

The accuracy of the clinical TRIAD consisting of symptoms in the dimensions of bowel and bladder dysfunction, orthopedic and neurological signs to define “occult filum terminale or occult tight filum syndrome” and the accuracy of the clinical TRIAD to predict surgical success of detethering has to be explored and proven in a prospective fashion.
